# Personalized Theta and Beta Binaural Beats for Brain Entrainment: An Electroencephalographic Analysis

**DOI:** 10.3389/fpsyg.2021.764068

**Published:** 2021-11-11

**Authors:** César E. Corona-González, Luz María Alonso-Valerdi, David I. Ibarra-Zarate

**Affiliations:** Escuela de Ingeniería y Ciencias, Tecnológico de Monterrey, Monterrey, Mexico

**Keywords:** binaural beats, beta, theta, EEG, brain entrainment

## Abstract

Binaural beats (BB) consist of two slightly distinct auditory frequencies (one in each ear), which are differentiated with clinical electroencephalographic (EEG) bandwidths, namely, delta, theta, alpha, beta, or gamma. This auditory stimulation has been widely used to module brain rhythms and thus inducing the mental condition associated with the EEG bandwidth in use. The aim of this research was to investigate whether personalized BB (specifically those within theta and beta EEG bands) improve brain entrainment. Personalized BB consisted of pure tones with a carrier tone of 500 Hz in the left ear together with an adjustable frequency in the right ear that was defined for theta BB (since *f*_*c*_ for theta EEG band was 4.60 Hz ± 0.70 SD) and beta BB (since *f*_*c*_ for beta EEG band was 18.42 Hz ± 2.82 SD). The adjustable frequencies were estimated for each participant in accordance with their heart rate by applying the Brain-Body Coupling Theorem postulated by Klimesch. To achieve this aim, 20 healthy volunteers were stimulated with their personalized theta and beta BB for 20 min and their EEG signals were collected with 22 channels. EEG analysis was based on the comparison of power spectral density among three mental conditions: (1) theta BB stimulation, (2) beta BB stimulation, and (3) resting state. Results showed larger absolute power differences for both BB stimulation sessions than resting state on bilateral temporal and parietal regions. This power change seems to be related to auditory perception and sound location. However, no significant differences were found between theta and beta BB sessions when it was expected to achieve different brain entrainments, since theta and beta BB induce relaxation and readiness, respectively. In addition, relative power analysis (theta BB/resting state) revealed alpha band desynchronization in the parieto-occipital region when volunteers listened to theta BB, suggesting that participants felt uncomfortable. In conclusion, neural resynchronization was met with both personalized theta and beta BB, but no different mental conditions seemed to be achieved.

## Introduction

In 1839, Heinrich Wilhelm Dove found that providing two slightly different tone frequencies, one in each ear, were perceived as a third phantom frequency depicted by the difference of these two frequencies, which was called binaural beats (BB; Keeley, 2006). It was until the 1950’s when Robert Monroe formally started to research the clinical application of BB, establishing that the dissimilarity of both frequencies must be within the electroencephalographic (EEG) spectrum, that brain entrainment could be elicited (Berger and Turow, 2011). Later, Worden and Marsh (1968) were investigating about electrophysiological effects of sound on the brain. They found that an auditory stimulus provokes a synchronous-neural evoked response which reproduces the frequency and waveform of the incoming stimulus throughout the central auditory pathway. This effect was coined as Frequency Following Response (FFR; Marsh and Worden, 1968; Worden and Marsh, 1968).

Neurons oscillate in several well-known EEG frequency bands. These are delta (δ = 0.1–4 Hz), theta (θ = 4–8 Hz), alpha (α = 8–13 Hz), beta (β = 13–30 Hz), and gamma (γ > 30 Hz). Normally, delta band is present while deep sleep; theta band is in extremely relaxation, drowsiness, or meditation; alpha is best seen at rest with eyes closed; beta is present during problem solving and focusing; and gamma is characterized by cognitive and motor functions (Siuly et al., 2016). In terms of EEG frequency bands, BB has a frequency difference within the band range of interest. For each EEG frequency band, the following corresponding BB can be generated: (1) delta BB, theta BB, alpha BB, beta BB, and gamma BB. In theory, each BB produces neural oscillations at the corresponding EEG frequency band, inducing the associated mental state. Tone frequencies stimuli between 450 and 500 Hz are recommended (García Argibay, 2018).

Theta and beta BBs are of particular interest since they may cause states of relaxation and attentiveness, respectively, which are opposite mental states so that they can be easily compared. In a study performed by Jirakittayakorn and Wongsawat (2017b), brain entrainment in theta wave was achieved when subjects listened to a 6 Hz BB for 10 min, promoting meditative states (Jirakittayakorn and Wongsawat, 2017b). Moreover, overwrought states due to insomnia were diminished by theta BB (Choi et al., 2019). In addition, beta BB has been used to improve (1) short-term (Gálvez et al., 2018) and long-term memory (García-Argibay et al., 2017), (2) working memory (Beauchene et al., 2016), (3) focusing levels and problem solution (Simmons, 2016), and (4) attention (Park et al., 2018). Conversely, López-Caballero and Escera (2017) disagree with brain entrainment due to BB, since no differences in EEG power between baseline and BB exposure were found while using theta, alpha, beta, gamma, and upper gamma BB (López-Caballero and Escera, 2017). Additionally, other studies failed on promoting brainwave entrainment using theta (Goodin et al., 2012; Orozco Perez et al., 2020) or beta BB (Goodin et al., 2012; Vernon et al., 2012). Another example is the work undertaken by Gao et al. (2014), where EEG signals were studied while delta, theta, alpha, and beta BB were applied. They did not find any brain entrainment after 20 min of BB stimulation (5 min per band, followed by a 2-min break between bands). Nevertheless, relative power variations within the four bands were thought to yield neural connectivity changes (Gao et al., 2014).

As shown by past studies, contradictory findings have been found. On one side, BB has shown to be successful in practice. On the other side, no EEG modulation (brainwave entrainment) has been achieved in all the BB studies. It is hypothesized that the BB effect can be always achieved if individual frequency bands of brain oscillations are found and used to generate BB. According to the Brain Body Coupling (BBC) theorem established by Klimesch (2018), brain and body (e.g., gastric waves, motion oscillations, blinks, and heart rate) oscillations are coupled to each other at rest (Klimesch, 2018). Therefore, individual brain-body frequency bands can be found if one of the brain-body oscillations is known, for example, heart rate (HR). On this basis, it is proposed to generate personalized theta and beta BB in accordance with individual theta and beta EEG frequency bands (previously found by BBC theorem), and then analyze the EEG modulation obtained after theta and beta BB exposure. For this purpose, the present investigation was undertaken as follows. First, 20 volunteers were recruited to whom audiometry was applied, and resting HR was taken (see section “Participants”). Second, theta and beta BBs were generated according to the HR of a participant (see section “Binaural Auditory Stimuli”). Third, the experiment was performed in two phases: (1) environment for binaural stimuli, where subjects were instructed about the experimental procedure (see section “Environment for Binaural Stimuli”) and (2) presentation of binaural stimuli, where EEG recordings were collected while participants were exposed to BBs (see section “Presentation of Binaural Stimuli”). Finally, EEG data analysis was carried out, which consisted in preprocessing (see section “Preprocessing”), processing (see section “Processing”), and statistical evaluation (see section “Statistical Evaluation”).

## Materials and Methods

### Participants

For this study, 20 healthy students of Tecnológico de Monterrey (six women and 14 men) aged between 19 and 24 years old were recruited (i.e., a convenience sampling method was undertaken). All of them reported not having musical experience and voluntarily consented to their participation in the study. This study was previously approved by the Ethical Committee of the Medicine School at Tecnológico de Monterrey (CONBIOETICA-19-CEI-011-20161017).

### Data Acquisition and Equipment

**FIGURE 1:**
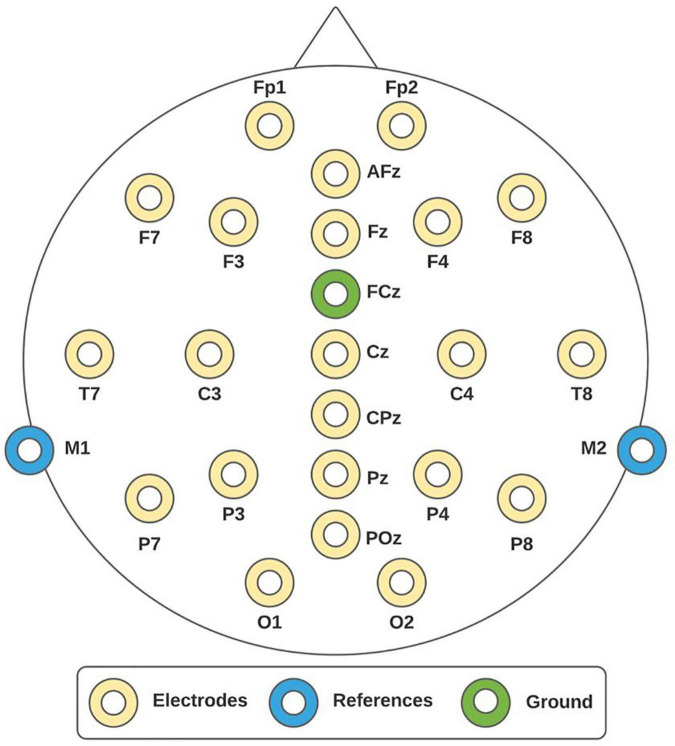
10/20 system. Electroencephalographic (EEG) montage consisted of 24 channels, whereby 22 of them were recording sites (yellow electrodes), one reference (blue electrodes), and one ground (green electrode).

To record EEG activity, the mBrainTrain system was used. This is a Bluetooth-interface EEG device of 24 channels (Fp1, Fp2, F3, F4, C3, C4, P3, P4, O1, O2, F7, F8, T7, T8, P7, P8, Fz, Cz, Pz, M1, M2, AFz, CPz, POz) positioned according to the 10/20 international system, as shown in Figure 1. The channels M1 and M2 were set as references and the channel FCz as ground. The sampling frequency was 250 Hz. The mBrainTrain has the Smarting Streamer software, which was used to verify electrode impedances to be below 5 kΩ. To set up the experimental paradigm, OpenVibe was employed. OpenVibe is a free software commonly used in the neuroscience field to design and test brain-computer interfaces and to develop experimental paradigms for offline records (Renard et al., 2010).

To perform the audiometry, an audiometer Interacoustics-AD226 was utilized. Tonal audiometry within ranges from 250 Hz to 2 kHz was used to determine auditory thresholds. Measurements were taken in dB HL. Finally, HR was taken with pulse oximeter Hergom-MD300 and auditory stimuli was given through open-back headphones SHURE-SRH1840.

### Binaural Auditory Stimuli

According to Klimesch (2013), biosignals do not vary randomly or arbitrarily. Namely, brain and body signals oscillations are aligned with each other and form a single frequency architecture. The interaction between brain and body may be described as a complex system that couples and decouples according to a specific harmony frequency described by,


(1) $$f_{d}\left(i\right)=s*2^{i}$$

where *s* is the scaling factor, *i* refers to the biosignal of interest, and *f* is the fundamental frequency of the biosignal oscillation. When *i* = 0, *f*_*d*_ refers to cardiac activity. When *i* < 0, *f*_*d*_ refers to breathing rhythms (including Mayer waves that are the lowest frequency in the respiratory process), blood pressure waves, rhythmic fluctuations in the blood oxygen level-dependent (BOLD) signal at intrinsic mode fluctuations, and gastric waves. When *i* > 0, *f*_*d*_ refers to brain oscillations [delta (*i* = 1), theta (*i* = 2), alpha (*i* = 3), beta (*i* = 4), gamma (*i* = 5)]. In addition, upper, and lower frequencies of each fundamental frequency can be, respectively, estimated by,


(2) $$uf_{b}\left(i\right)=\frac{1.25\times 2^{i+1}}{g}$$

(3) $$lf_{b}\left(i\right)=\left(1.25\times 2^{i-1}\right)\times g$$

Recently, Klimesch (2018) empirically demonstrated that the resonance of a biosignal is harmonized with other ones at resting state. Some examples are:

• During respiration, HR increases at inhalation and decreases at exhalation.
• Heart rate presents a clear tendency 10:1 frequency ratio relative to breathing rate owing to energy demands and emotional regulation.
• Gastric waves explain 8% of alpha band modulation of EEG signals, and 15% of the BOLD variance is explained by the gastric phase.
• The slow frequency that modulates the envelope of the electromyographic signals is originated from neural mechanisms of motor control and resonance frequency of body parts.

In the light of the above evidence, this research proposes the design of personalized BB based on individual HR. That is, when *i* = 0 in Eq. (1) then,


$$f_{d}\left(0\right)=s*2^{0}=s=HR\left[Hz\right]$$

Having estimated *s*, central frequency (*f*_*c*_) of theta and beta EEG rhythms were calculated in accordance with the individual HR at hand. Note that resulting fundamental frequencies were not subjective, they were rather relative to the human body function, and their values are around the well clinically established frequency bands.

As theta BB consisted of pure tones of 500 Hz for the left ear, *f*_*c*_ of theta EEG band was used to adjust the frequency for the right ear (*F*_*R_ear*_). Similarly, beta BB consisted of pure tones of 500 Hz for the left ear. Then, *f*_*c*_ of beta EEG band was used to adjust the frequency for the right ear. That is,


(4) $$F_{R\_ear}=500Hz-f_{c}$$

The resulting EEG frequency bands for each volunteer, and the corresponding BB produced after the individual EEG frequency band identification, are reported in Table 1. The computational algorithm to generate personalized BB in accordance with the BBC theorem was programmed in MATLAB programming language and was published in MathWorks File Exchange Forum^1^.

**TABLE 1:** Volunteer data.

**Volunteer**	**EEG fundamental frequency (*f*_*c*_)**		**Theta BB (S1)**		**Beta BB (S2)**	
	**Theta (Hz)**	**Beta (Hz)**	**Left (Hz)**	**Right *F*_*R_ear_*_ (Hz)**	**Left (Hz)**	**Right *F*_*R_ear_*_ (Hz)**
1	4.33	17.33	500	495.67	500	482.67
2	4.8	19.2	500	495.2	500	480.8
3	4	16	500	496	500	484
4	3.87	15.47	500	496.13	500	484.53
5	5.4	21.6	500	494.6	500	478.4
6	4.2	16.8	500	495.8	500	483.2
7	5.13	20.53	500	494.87	500	479.47
8	4.67	18.67	500	495.33	500	481.33
9	5.93	23.73	500	494.07	500	476.27
10	4.73	18.93	500	495.27	500	481.07
11	4.67	18.67	500	495.33	500	481.33
12	5.93	23.73	500	494.07	500	476.27
13	3.87	15.47	500	496.13	500	484.53
14	4.53	18.13	500	495.47	500	481.87
15	4.73	18.93	500	495.27	500	481.07
16	3.2	12.8	500	496.8	500	487.2
17	4	16	500	496	500	484
18	5.53	22.13	500	494.47	500	477.87
19	4.87	19.47	500	495.13	500	480.53
20	4.73	18.93	500	495.27	500	481.07
Mean	**4.60**	**18.42**	Mean	**495.34**	Mean	**481.37**
S.D	**0.70**	**2.82**	S.D	**0.70**	S.D	**2.82**

*f_c_ for theta and beta bands and F_R_ear__ for BB customizing for S1 and S2.*

### Experimental Paradigm and Protocols

Each volunteer participated in two BB sessions on different days. In the first session (S1), theta BB was applied, and in the second session (S2), beta BB was used. Both BB exposures were for 20 min, since listening to BB longer than 20 min may lead to mental fatigue (Jirakittayakorn and Wongsawat, 2017a). All the participants were seated on a comfortable chair and in a quiet room. All of them were asked to keep their eyes closed during BB stimulation. The procedure was conducted in two steps: (1) environment for binaural stimuli and (2) presentation of binaural stimuli.

#### Environment for Binaural Stimuli

**FIGURE 2:**
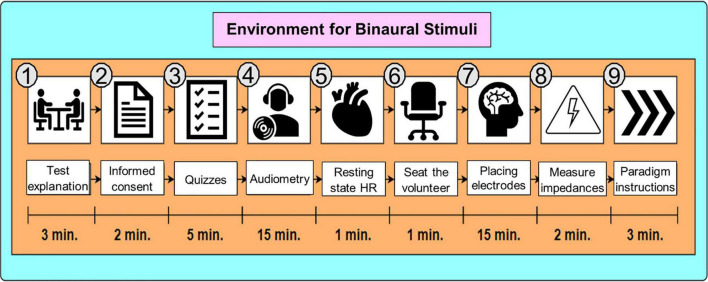
Environment for Binaural Stimuli. The preparation stage in S1 was about 47 min long. For S2, it was about 21 min long since the procedure started at step 6 (sitting down the volunteer).

First, the purpose of the study was explained to the participant, and after agreeing to their participation, they signed a consent form. Second, the Neurologic Evaluation Questionnaire from Neuroscience Institute of University of Guadalajara (Balart Sánchez, 2017) was applied to assess their medical history about neurological health. Third, tonal audiometry and the HR at resting state were taken. Audiometry and HR per volunteer are reported in Table 2. Fourth, the volunteer was asked to sit down and relax while putting on the EEG cap and electrodes impedances were controlled to be kept below 5 *k*Ω. Finally, paradigm instructions were given to the participant. Figure 2 shows the whole preparation sequence. For S2, volunteer preparation started from step 6 in Figure 2.

**TABLE 2:** Heart rate (HR) and audiometry values per volunteer.

**Volunteer**	**HR**	**Right ear (dB)**						**Left ear (dB)**					
		**250 Hz**	**500 Hz**	**750 Hz**	**1 kHz**	**1.5 kHz**	**2 kHz**	**250 Hz**	**500 Hz**	**750 Hz**	**1 kHz**	**1.5 kHz**	**2 kHz**
1	65	25	10	15	0	0	0	10	10	10	5	0	0
2	72	15	10	10	5	5	0	20	10	10	5	0	0
3	60	15	10	10	10	0	5	10	10	10	5	5	5
4	58	25	20	15	15	10	5	20	15	20	15	10	15
5	81	10	10	5	5	10	10	10	5	5	0	5	5
6	63	20	5	10	5	10	5	10	10	5	5	5	0
7	77	10	5	5	10	0	0	5	5	5	0	0	5
8	70	10	15	15	10	5	5	5	10	10	10	5	5
9	89	20	10	15	10	10	5	10	5	10	5	5	0
10	71	5	15	10	0	0	0	15	20	15	5	0	0
11	70	10	15	15	10	5	5	15	15	15	10	5	5
12	89	20	15	10	15	10	5	15	5	10	10	5	0
13	58	10	5	10	10	5	0	0	5	5	5	0	0
14	68	5	0	0	0	5	0	0	5	5	0	0	0
15	71	10	5	5	5	5	0	15	5	5	0	5	0
16	48	15	10	10	5	10	5	20	10	10	5	5	0
17	60	5	5	10	5	10	0	10	5	5	0	0	0
18	83	30	25	15	10	5	5	25	15	20	10	5	0
19	73	5	5	10	5	10	0	10	5	5	0	0	0
20	71	15	10	10	5	10	5	10	10	10	5	10	0

*HR was taken at rest in each volunteer, whereas audiometry was performed from 250 Hz to 2 kHz.*

#### Presentation of Binaural Stimuli

First, volunteers were instructed to keep their eyes closed for 3 min. Hereinafter, this EEG recording is referred to as to baseline or resting state session (S_*R*_). Second, volunteers were stimulated with their personalized theta and beta BB for 20 min in S1 and S2, respectively. The sound level was 60 dB SPL for both sessions (World Health Organization [WHO] and International Telecommunication Union [ITU], 2019).

### Signal Analysis

Power spectral density is a method to extract the power content of a signal in the frequency domain. Power spectral density (PSD) utilizes the Discrete Fourier Transform to obtain the periodogram. Welch’s method is one of the most applied algorithms to estimate PSD (Zhang, 2019). In this study, PSD was calculated for the three different mental states: (1) S_*R*_, (2) S1, and (3) S2. The analysis was carried out in two steps: (1) preprocessing and (2) processing.

#### Preprocessing

**FIGURE 3:**
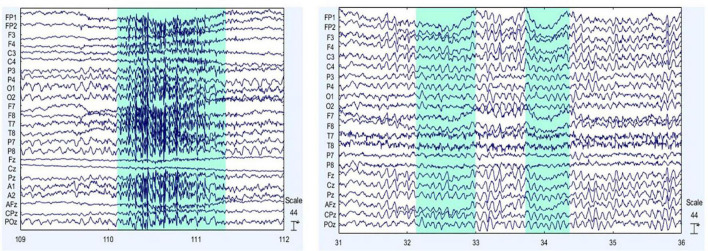
Main EEG artifacts are ocular and muscular electrical activity. On the left **(A)**, muscular artifacts are shown, and on the right **(B)**, ocular artifacts are presented. The sections shaded in blue indicate examples of the muscular (on the left) and eye (on the right) artifacts themselves.

Electroencephalographic signals were preprocessed in MATLAB using the EEGLab toolbox, developed by the Swartz Center for Computational Neuroscience at the University of California, San Diego (Martínez-Cancino et al., 2020). For preprocessing, the sampling frequency was 250 Hz, the Direct Current component was removed, and a bandpass filter from 0.1 to 100 Hz was utilized along with a band-stop filter for removing 59–61 Hz. Both filters were IIR Butterworth 8th order. FCz was set as the ground electrode and re-referencing was regarding M1 and M2 average. Then, visual inspection was required for cleaning up EEG signals from abrupt changes due to muscular artifacts. Finally, Independent Component Analysis was applied for ocular and cardiac artifacts removal. Figures 3A,B exemplify the muscular and ocular artifacts of one of the volunteers. The raw EEG data set is freely accessible and is available in the Mendeley database at https://data.mendeley.com/datasets/ppz3r5j2n2/2.

#### Processing

**FIGURE 4:**
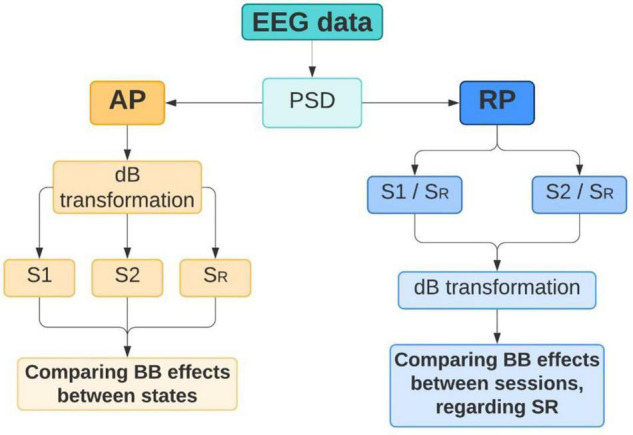
Power spectral density (PSD) analysis. Absolute power (AP) and relative power (RP) were extracted from EEG data in order to identify brain changes that can denote brain entrainment.

To quantify BB effects, PSD was extracted from all volunteers in S_*R*_, S1, and S2 conditions. For Fourier transform algorithms, such as Welch’s method, stationarity must be satisfied. However, EEG signals can be segmented into short windows where stationarity is assumed (Nunez et al., 2016), especially when emotional processes are mediated by visual or audio-visual stimuli (Aydın, 2020). For that reason, PSD was applied to each volunteer dataset using a windowing of 1 s and an overlapping of 50%. The analysis was carried out taking into consideration two parameters from PSD: (1) absolute power (AP) from S_*R*_, S1, and S2; and (2) relative power (RP) from S1/S_*R*_ and S2/S_*R*_. Analyzing AP can provide spectral information regarding neural activity before and after listening to theta or beta BB (Park, 2020). Thus, brain entrainment can be identified in S1 if theta BB triggers the highest AP in theta EEG band. Similarly, brain entrainment can be detected for S2 if beta BB elicits the highest AP in the beta EEG band. However, as comparing AP for the three mental states, it is difficult to differentiate precise changes in EEG frequencies. Therefore, using RP for S1/S_*R*_ and S2/S_*R*_ allows to directly compare the influence of both theta and beta BB over resting state, so that brain entrainment can be supported. Figure 4 summarizes these two analyses.

##### Power Spectral Density – Absolute Power

Power spectral density was calculated for S_*R*_, S1, and S2 to obtain the AP of each mental state. AP values were allocated in three different matrices (one for each state), which dimensions refer to volunteers (20) by channels (22) by AP values (126). Then, these matrices were averaged by volunteers and transformed into a decibel (dB) scale. As the highest frequencies of interest are in the beta range, only frequencies from 0 to 30 Hz were considered for the analysis. Finally, power values within these frequencies were compared in every channel for S_*R*_, S1, and S2.

##### Power Spectral Density – Relative Power

Once PSD for S_*R*_, S1, and S2 was individually estimated, a data standardization was performed to the power values of S1 and S2, both regarding S_*R*_. The standardized value represents the Relative Power (RP) between BB session and S_*R*_. Eq. (5) summarizes the calculation of RP:


(5) $$RP_{s}^{v}=AP_{s}^{v}/AP_{R}^{v}$$

where,

$RP_{s}^{v}$ represents the relative power of volunteer “v” and session “s” (theta BB or beta BB).
$AP_{s}^{v}$ is AP from PSD of volunteer “v” of session “s” and
$AP_{R}^{v}$ is AP from PSD of volunteer “v” at resting state.

It should be noted that Eq. (5) was applied to every channel. Therefore, RP for every volunteer across all channels has been calculated until now. After that, two averaged-by-volunteer matrices were computed, one for S1/S_*R*_ and the other for S2/S_*R*_. Finally, dB transformation was applied. These ratios may manifest the power variation on theta, alpha, and beta bands and may confirm if brain entrainment has been achieved for the specific band (i.e., increase in theta band activity due to theta BB or increase in beta band activity due to beta BB). Owing to the wide frequency range of the gamma band, it was excluded from this analysis. Considering that the theta band was the minimum frequency range for inducing the FFR effect, the delta band was also rejected for the analysis.

Maximum power in theta and beta EEG bands was expected when volunteers were listening to theta and beta BBs, respectively. Therefore, changes in magnitude power of EEG data could be seen and brain entrainment may be accomplished.

### Statistical Evaluation

Statistical analysis was carried out for AP and RP estimates. With respect to AP estimates, the comparison was between sessions (1) S_*R*_ with S1, and (2) S_*R*_ with S2. First, the Shapiro–Wilk test for normality (Ahad et al., 2011) was applied for these two pairs of data. Once normality was confirmed for both cases, a separate *t*-test was performed to find significant differences between S_*R*_-S1 and S_*R*_-S2 through all channels. Afterward, Cohen’s d effect size was utilized to estimate the magnitude of these differences as “negligible” (*d* < 0.2), “small” (0.2 ≤ *d* < 0.5), “medium” (0.5 ≤ *d* < 0.8), or “large” (*d* ≥ 0.8) (Magnusson, 2021). Regarding RP estimates, normality was also tested using the Shapiro–Wilk test for the power difference between theta, alpha, and beta bands in both S1 and S2 in every channel. Subsequently, two alternately two-way ANOVA tests were performed to RP in S1 and S2. The tested values included averaged RP values, channels, and bands. These two ANOVAs were aimed to verify if statistically significant changes in RP amongst EEG bands and channels were achieved after BB stimulation. Finally, a Tukey test was realized (Daniel and Cross, 2013) to locate these differences. A significance level of 0.05 was used in all statistical tests.

## Production of Theta and Beta Binaural Beats Sound

Theta and beta BB were designed in MATLAB at .*wav* format, with a sampling frequency of 44,100 Hz (Pejrolo and Metcalfe, 2017). BB had an amplitude-modulated sound composed of two pure frequency tones. According to HR estimations shown in Table 2, *f*_*c*_ for each EEG band was calculated based on BBC theorem (Klimesch, 2018). By way of illustration, assuming an HR of 70 bpm, *s* turns out as:


$$70/60=1.16\bar{6};$$

From Eqs (1) and (4), it is explained the mathematical procedure for individual *f*_*c*_ for theta and beta bands, and *F*_*R_ear_*_ for the personalized design of BB routines, respectively. These values are calculated as follows:

• Theta band:

$$f_{c}=1.16\bar{6}*2^{2}=4.67Hz$$

$$F_{R_{ear\theta }}=500Hz-4.67Hz=495.33Hz$$

• Beta band:

$$f_{c}=1.16\bar{6}*2^{4}=18.67Hz$$

$$F_{R_{ear\beta }}=500Hz-18.67Hz=481.33Hz$$

Hence, stimulation frequencies to create theta BB were 500 and 495.33 Hz and for beta BB were 500 and 481.33 Hz. Table 1 shows *f*_*c*_ and *F*_*R_ear*_ values of all volunteers.

## Results

Twenty volunteers aged between 19 and 24 were recruited, who reported normal hearing thresholds, good neurological history, and no musical experience. Before S1 was performed, S_*R*_ was taken as the baseline for 3 min. Afterward, S1 and S2 lasted 20 min each. The data analysis was based on AP and RP of EEG signals within 0 and 30 Hz. Findings are mentioned below.

### Comparison Between Theta and Beta Binaural Beats Effects: Absolute Power Estimation

**FIGURE 5:**
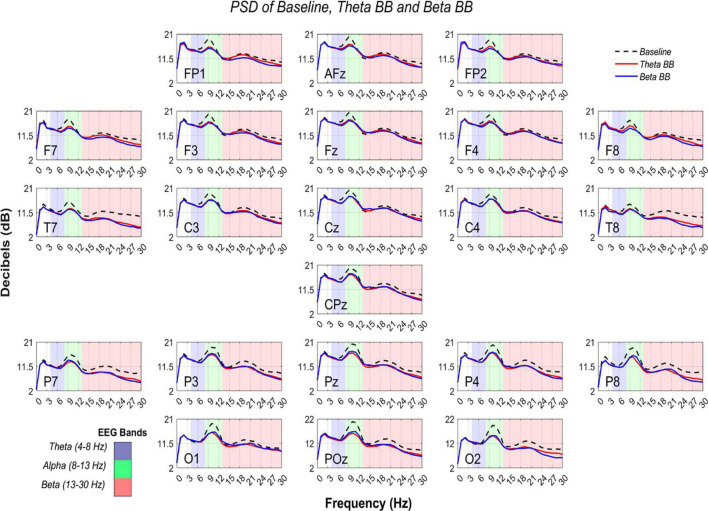
Comparison of PSD from S1, S2, and S_*R*_. AP in dB (*Y*-axis) are shown from 0 to 30 Hz (*X*-axis). Black dashed line represents average AP from S_*R*_, whereas solid lines depict average AP from theta binaural beats (BB) (red) and beta BB (blue). The colored background separates theta (blue), alpha (green), and beta (red) bands.

Absolute power from S1 and S2 were compared with S_*R*_ to identify if BB exposure elicited changes in neural activity. In Figure 5, it is exhibited average AP from the three conditions in dB within the frequency range of 0–30 Hz. PSD was estimated across all channels.

**FIGURE 6:**
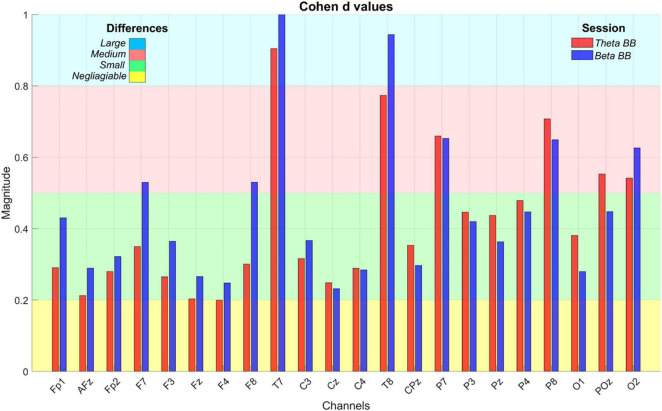
Cohen’s d effect size values. The bar plot depicts Cohen’s *d* values to estimate the magnitude of statistical differences in absolute power when S1 (red bar) and S2 (blue bar) were compared with S_*R*_. *X*-axis exhibits EEG channels whereas *Y*-axis is the Cohen’s *d* value, ranging from 0 to 1. The colored background regions represent the intervals to specify the magnitude of the differences throughout channels and between sessions, such as negligible (yellow), small (green), medium (red), and large (blue).

At an initial glance, it seemed that average S1, S2, and S_*R*_ triggered similar brain activity. To dismiss this issue, a paired *t*-test was applied to AP values of (1) S1 with S_*R*_ and (2) S2 with S_*R*_, for all channels. Significant differences were found across all the channels in both comparisons (*p* < 0.05). However, a Cohen’s d effect size test was implemented to estimate the magnitude of these differences. Cohen’s *d* values are graphically expressed in Figure 6.

### Comparison Between Electroencephalographic Frequency Bands: Relative Power Estimation

**FIGURE 7:**
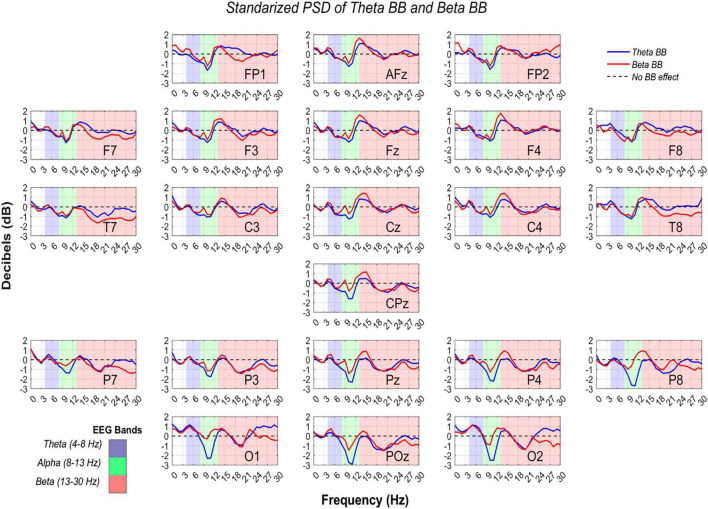
Power spectral density from theta and beta BB, regarding resting state. RP values from theta BB (blue solid line) and beta BB (red solid line) are shown, where *X*-axis is depicted by frequency ranging from 0 to 30 Hz and *Y*-axis is the averaged power ratio between S1/S_*R*_ and S2/S_*R*_ in dB. Every plot represents a different channel, which was labeled in the lower-right corner. Measurements are highlighted in blue for the theta band, in green for the alpha band, and in red for the beta band.

In order to confirm if brain entrainment was achieved, the following conditions must be met: (1) for theta BB, an increase in S1/S_*R*_ ratio in the theta band, or (2) for beta BB, an increment in S2/S_*R*_ ratio in the beta band. Thus, RP from S1/S_*R*_ and S2/S_*R*_ were compared across theta, alpha, and beta bands. Figure 7 shows RP from all channels in dB, throughout EEG frequency ranges where theta, alpha, and beta were colored in blue, green, and red, respectively.

So far, it is known that statistical differences in all channels were identified between BB sessions and resting state, but it does not confirm if brain entrainment was attained. For this reason, a two-way ANOVA was consecutively applied to RP from S1 and S2 to verify if neural activity between bands were dissimilar due to BB. The ANOVA data for each matrix contained RP values from S1 or S2, channels, and bands (theta, alpha, and beta). Significant differences were found for alpha-theta bands (*p* = 0) and alpha-beta bands (*p* = 0). Nevertheless, no statistical difference was obtained for theta-beta (*p* = 0.5577).

## Discussion

This study was focused on investigating if brain entrainment could be achieved from the modulation of EEG signals by personalized theta and beta BB stimulation. The EEG analysis followed two approaches: (1) AP, obtained from individual PSD of S1, S2, and S_*R*,_ and (2) RP, computed by S1/S_*R*_ and S2/S_*R*_ power ratios. In the following, the results of this research are discussed for each method.

### Absolute Power Estimation

As can be seen from Figure 6, T7 and T8 showed the highest differences when BB sessions were compared against S_*R*_, followed by medium differences in P7, P8, and O2. After listening to either theta or beta BB, small to medium changes in AP occurred in the remaining channels, suggesting that significant effects due to BB were not entailed or, in other words, brain entrainment was not achieved. As the greatest changes were seen over the temporal lobe, followed by the parietal one, they may be associated with auditory perception and sound location (Benarroch, 2006; Bizley and Cohen, 2013; Goldstein, 2014).

### Relative Power Estimation

Since data standardization was carried out, a 0 dB value means that BB session and baseline neural activity were comparable. On the contrary, when dB > 0, brain activity was stronger during BB exposure than baseline. Thus, either theta or beta BB elicited neural synchronization. Similarly, given a dB < 0, it implies that brain activity was higher in the resting state in comparison with the BB session, which means that neural desynchronization was induced (Watkinson and Clarke, 2018).

According to Cohen (2014), an oscillation from −2 dB to +2 dB implies a percentage change from −36.9 to +58.8%. In other words, if RP was equal to −2 dB, it means that baseline activity was higher than in BB session by 36.9%, (i.e., BB induced 36.9% of neural desynchronization). Moreover, a +2 dB change means that the BB session had stronger brain activity than baseline by 58.8% (i.e., BB triggered 58.8% neural synchronization) (Cohen, 2014).

Interestingly, RP in the parieto-occipital region was lesser than −2 dB for alpha EEG band while listening to theta BB, specifically over Pz, P4, P8, O1, POz, and O2 recording sites. This decrease in dB value is explained by greater brain activity at the resting state regarding theta BB. For these channels, as alpha desynchronization occurred only for S1, we suggest that theta BB probably disturbed volunteers instead of inducing them into meditative or relaxed states, even though when they were just seated with closed eyes and doing no task. A theory of this behavior may be related to volunteers feeling uncomfortable when listening to the theta BB (Crespo et al., 2013; Lee et al., 2019).

In conclusion, neural resynchronization was met with both personalized theta and beta BB, but no different mental conditions seemed to be achieved.

### Limitations of the Study

The aim of the study was mainly limited due to (1) conditions of participants, and (2) the use of open-back headphones. First, for more specificity in sample selection, hours of sleep in the night before the study and psychological conditions, such as stress or anxiety, should have been considered. These factors can disturb brain oscillations. Second, as BB was delivered through open-back headphones, the environment auditory stimuli could bias neural information. Therefore, the study should have been implemented in an isolated room.

### Future Work

In order to develop a full picture of brain activity due to BB exposure, a suggestion would be that the application of other kinds of BB not used in this study such as alpha BB (Park et al., 2018; Shekar et al., 2018) or gamma BB (Colzato et al., 2017; Shekar et al., 2018). Additional studies that give an insight into brain signal modulation are needed for further understanding of which effects BB induces on humans. Empirical studies where BB effects are behaviorally measured are not enough to demonstrate binaural sound influence on the human mental state.

## Data Availability Statement

The original contributions presented in the study are publicly available. This data can be found here: https://data.mendeley.com/datasets/ppz3r5j2n2/2.

## Ethics Statement

The studies involving human participants were reviewed and approved by the Ethical Committee of the Medicine School at Tecnológico de Monterrey (CONBIOETICA-19-CEI-011-20161017). The patients/participants provided their written informed consent to participate in this study.

## Author Contributions

CC-G contributed to the design of methodology, programming software for signal acquisition, signal processing, statistical analysis, and writing the original draft. LA-V and DI-Z served as advisor of the research by methodology design, feedback on data analysis and statistical analysis, and review and editing the manuscript. All authors contributed to the manuscript revision, read and approved the submitted version.

## Conflict of Interest

The authors declare that the research was conducted in the absence of any commercial or financial relationships that could be construed as a potential conflict of interest.

## Publisher’s Note

All claims expressed in this article are solely those of the authors and do not necessarily represent those of their affiliated organizations, or those of the publisher, the editors and the reviewers. Any product that may be evaluated in this article, or claim that may be made by its manufacturer, is not guaranteed or endorsed by the publisher.

## Footnotes

1
https://www.mathworks.com/matlabcentral/fileexchange/99544-personalized-binaural-beats-generator

## Abbreviations

AP: Absolute power
BB: Binaural beats
BBC: Brain Body Coupling
BOLD: Blood Oxygen Level Dependent
dB HL: Decibels hearing level
EEG: Electroencephalography
f_c_: Central frequency
F_R_ear__: Right ear stimulation frequency
HR: Heart rate
PSD: Power spectral density
RP: Relative power
S1: Session 1 (theta BB)
S2: Session 2 (beta BB)
SPL: Sound pressure level
S_*R*_: Resting state session.
